# Treatment Preferences in Acute Psychosis: A Comparison of Patient and Staff Perspectives on Symptom Prioritization and Biopsychosocial Interventions

**DOI:** 10.1007/s11126-024-10099-2

**Published:** 2024-10-29

**Authors:** Rabea Fischer, Steffen Moritz, Jakob Scheunemann, Matthias Nagel, Charlotte Osthues, Daniel Schöttle, Daniel Luedecke

**Affiliations:** 1 Department of Psychiatry and Psychotherapy, University Medical Center Hamburg-Eppendorf, Hamburg, Germany; 2 Department of Psychiatry and Psychotherapy, Asklepios Clinic North-Wandsbek, Hamburg, Germany; 3 Clinic for Psychiatry and Psychotherapy, University Clinic Schleswig-Holstein, Campus Luebeck, Luebeck, Germany; 4 Department of Psychiatry and Psychotherapy, Asklepios Clinic Harburg, Hamburg, Germany

**Keywords:** Locked ward, Schizophrenia, Bipolar Disorder, Severe Mental Illness, Psychological Intervention

## Abstract

Patients with acute psychosis are often confronted with the prejudice that they refuse all treatment due to lack of insight. This study examined and compared the aims and preferences for treatment of patients with acute psychosis and of psychiatric inpatient staff. A total of 142 inpatients being treated for a psychotic disorder on either a locked or an open ward indicated which of a range of symptoms they want to be treated and which of various biopsychosocial treatment options they would like to receive. Staff members from the same wards reported which psychiatric symptoms they deemed relevant in the treatment of psychosis and which treatment options should be offered. Patients assigned the highest treatment need to neurocognitive symptoms, followed by affective and positive symptoms. In contrast, staff assigned the highest treatment need to positive symptoms, followed by neurocognitive and affective symptoms. Patients and staff on open wards expressed more treatment needs overall than did patients and staff on locked wards. Patients’ desire for treatment differed across treatment types. In comparing patients on locked versus open wards, patients on open wards expressed higher approval of medication than patients on locked wards. Even patients with highly acute psychosis being treated on locked psychiatric wards endorsed treatment. Treatment preferences of this group deviated markedly in some instances from staffs’ preferences. Considering their specific needs may contribute to increasing patient self-efficacy, and improving adherence to treatment.

**Supplementary Information:**

The online version contains supplementary material available at 10.1007/s11126-024-10099-2.

## Introduction

Psychotic disorders, especially schizophrenia and bipolar disorder with psychotic features, are considered severe mental illnesses that often take a chronic course. Both schizophrenia and bipolar disorder are associated with reduced psychosocial functioning, somatic comorbidities, and increased mortality [1–5]. People diagnosed with psychotic disorders are particularly likely to experience acute psychiatric treatment, and a diagnosis of a psychotic disorder is a strong risk factor for involuntary hospitalization [6].

Psychiatric treatment’s emphasis on biological processes and its relationship to complementary services such as psychological interventions have at times been the subject of heated discussions [7]. In contrast to the traditional clinical definition of remission as symptom reduction [8], the patient-centered recovery movement that emerged in the late 20th century has focused on overall well-being, independent of (at times persistent) symptom presence [9, 10]. Within this context, psychosocial functioning and subjective quality of life constitute some of the main outcomes. Research has shown an association between symptom severity and subjective measures of recovery [11]. Nevertheless, patients’ self-rated needs for care, which must be met to be able to reach certain recovery goals, contribute to predictions of subjective quality of life, and to perceived psychosocial disabilities beyond psychopathology [12].

Although not all such needs apply to the acute psychiatric care setting, some, such as general psychological distress reduction, may be addressed in this context. Particularly in inpatient care for psychosis, however, the emphasis of patients and staff regarding treatment goals can differ [13]. Staff may primarily aim for quick stabilization through medication and risk containment, leaving little room for psychological approaches [14]. Patients, on the other hand, want more psychosocial treatment options, better patient-staff relationships, and more involvement in their treatment plan, including a focus on reducing the symptoms they view as distressing [13]. These differences may in part be explained by different models and conceptualizations of mental health and illness held by patients and staff [15, 16].

Patients’ reluctance, or at times even refusal, to treat certain symptoms, particularly positive symptoms, is commonly defined as a subdomain of insight into illness and therefore as a symptom to be treated in itself [17]. Yet, research shows that even patients who have less acute symptoms than those on acute wards and who actively seek treatment view some of their positive symptoms favorably and want them to remain present [18, 19]. In studies asking outpatients with schizophrenia to indicate the treatment importance of a range of symptoms, patients assign more importance to affective and neurocognitive symptoms than to positive symptoms, whereas physicians assign the most importance to neurocognitive symptoms [20, 21]. Whether these findings also apply to inpatients with severe acute symptoms (and whether these patients see a need for treatment at all) remains to be shown.

Overall, acute psychiatric inpatient care must strive to improve and broaden its treatment approach to include more psychosocial options, which patients have expressed a need for [22, 23]. While several studies have shown promising results for psychological interventions in acute settings [24], the willingness of patients with severe acute exacerbations of psychotic symptoms to engage in various treatment options within a biopsychosocial treatment framework remains under-researched. Staff working in an acute setting may hesitate to recommend patient participation in psychosocial interventions because, for instance, they question patients’ ability to understand the interventions’ aims [25].

We hypothesized that patients on acute wards would report experiencing a variety of symptoms, including affective, neurocognitive, and positive symptoms, and that they would assign a higher need for treatment to affective and neurocognitive than to positive symptoms. In addition, we hypothesized that patients would want a variety of psychosocial treatment options and that some patients, while generally open to receiving treatment, would not wish for pharmacological treatment. We hypothesized that staff would assign a higher need for treatment to positive and neurocognitive symptoms than to affective symptoms and that they would endorse patients receiving a variety of psychosocial treatment options as well as pharmacological treatment. Concerning differences between the open and locked wards, we hypothesized that both patients and staff on open wards would report more treatment need than patients and staff on locked wards. As for treatment options, we hypothesized that, overall, patients on locked wards would not differ from patients on open wards regarding endorsement of psychosocial treatment options but that staff on locked wards would be more hesitant to endorse certain psychosocial treatment options than staff on open wards.

## Materials and Methods

### Design

We conducted a survey of patients with psychotic disorders on acute locked vs. open psychiatric wards. Patients completed questionnaires regarding their prioritized treatment targets (symptoms) as well as their preferences regarding their upcoming treatment on their ward. Prior to participation, all patients gave written informed consent. In a parallel design, staff on these wards filled in analogous questionnaires regarding their own perceived priorities in treating their patients. The University Medical Center Hamburg-Eppendorf’s Ethics Committee for Psychological Studies approved the study (LPEK-0152), which was carried out in accordance with the ethical standards laid down in the 1964 Declaration of Helsinki and its later amendments.

### Setting

The study was conducted at two sites: the Department of Psychiatry and Psychotherapy of the University Medical Center Hamburg-Eppendorf (UKE) and the Department of Psychiatry and Psychotherapy of the Asklepios Clinic North-Wandsbek (AKNW), Germany. The UKE has two locked wards with 13 and 19 beds respectively, and one open ward with 23 beds specifically for patients with psychosis and/or bipolar disorder. The AKNW has two locked wards with 21 beds each and one open ward specifically for patients with psychosis and/or bipolar disorder with 26 beds. All locked wards provide intensive psychiatric care for patients with a variety of psychiatric diagnoses who pose potential harm to themselves or others due to their medical condition. Patients on the locked wards are generally more acutely ill than those on the open wards, and many are in mandatory treatment. The two hospitals’ catchment sectors are urban areas with approximately 450,000 and 320,000 residents, respectively.

### Sample

Patients were eligible for participation if they had a primary diagnosis of any psychotic disorder, including but not limited to, schizophrenia spectrum disorders (as classified in the DSM-V or the ICD-10 F-section), were currently undergoing inpatient treatment on one of the six wards, and were at least 18 years old. Exclusion criteria were inability to consent, insufficient German language skills, intellectual disability, dementia, and inability to obtain consent from a legal guardian when applicable.

All professional staff were invited to participate in this survey if they were currently working on one of the six wards patients were recruited from.

### Procedure

Patients were consecutively recruited shortly after admission to the inpatient wards (although in some cases patients were recruited later due to high symptom burden upon admission). A total of 1,985 admission records were screened for potential participants (see Figure S1 in Online Resource 1). Patients provided written informed consent to participate in the study and then completed the interview. The interview length varied depending on symptom severity but averaged 20 to 30 min.

#### Instruments

Parallel questionnaires regarding symptom treatment priorities and treatment type priorities were devised for patients and staff. Both were accompanied by sociodemographic questions.

Patients were interviewed using a face-to-face paper-pencil format; staff filled in an online survey they received via email.

The patient questionnaire included one open-ended question regarding patients’ perception of current symptoms (“Which problems/symptoms are you currently experiencing that you wish to receive treatment for during your stay on this ward?”). After the open-ended question, patients were asked the following question regarding a list of 16 symptoms: “Are you currently experiencing the following symptom or problem?” If they answered yes, they were asked “Would you like treatment or help with this problem while you are on this ward?” Answers were given on a five-point scale (definitely yes, somewhat yes, unsure, somewhat no, definitely no). Some symptoms were described rather than named directly (e.g., “Is there anything special about you? Do you have any special abilities or powers?” for grandiosity). Whenever a patient did not endorse experiencing a given symptom, they were not asked about it further.

In addition, the patient questionnaire included two open-ended questions regarding treatment preferences (“Which types of treatment do you expect to receive on this ward?” and “Which types of treatment would you like to receive on this ward?”). Following the open-ended questions, patients were asked about eight types of treatments for psychosis. First, they were asked whether they were familiar with the given treatment (meaning they knew what this treatment is; it was not necessary for them to have experienced the treatment themselves). If they said yes, then they were asked whether they would like to receive that treatment on the ward they were currently on. Responses were recorded using a three-point scale: yes, unsure, no.

The staff questionnaire posed questions analogous to the patient questionnaire, including one open-ended question regarding symptoms (“In your opinion, which symptoms typically experienced by individuals with psychosis do you consider most crucial to address in your ward setting?”). It also inquired about the same 16 symptoms patients were asked about (“Do you believe that the following symptoms/difficulties, if exhibited by a patient, should be treated in your ward setting?”), using the same five-point scale as on the patients’ questionnaire.

The staff questionnaire also included an open-ended question on treatments (“What types of treatment do you think should be offered to individuals with psychosis on your ward?”), followed by a question about the eight treatment types mentioned previously: “Which of the following types of treatment do you think should be offered to individuals with psychosis on your ward?” Responses were recorded using the same three-point scale as on the patients’ questionnaire.

### Data Analysis

Patients’ symptom presence, subjective need for treatment for a given symptom, and treatment type preferences, as well as staff’s views on symptom and treatment type importance, were analyzed descriptively. In addition, we clustered symptoms into subscales, such as positive or affective symptoms, based on theoretical assumptions. We carried out repeated measures ANOVAs using these symptom subscales as within-subject factor and ward setting as between-subject factor to investigate whether patients and/or staff assigned different treatment importance to various symptoms and whether this varied between the open and the locked ward setting. For the analysis of the patient sample, we also included gender as a between-subject factor to test for gender differences. In addition, we performed repeated measures ANOVAs using treatment type as within-subject factor and open versus locked setting (as well as gender for patients) as between-subject factor(s) to assess differences in patients’ and/or staff’s endorsement of different treatments between settings (and genders).

As not all patients experienced every symptom and not all were familiar with all the treatments on the questionnaire, we performed the ANOVAs using only the three most frequently present symptom subscales that were named by at least 100 patients each (positive, affective, and neurocognitive symptoms) and using only six of the eight treatments that more than 75% of the patient sample knew about, namely art therapy, group psychotherapy, individual psychotherapy, medication, occupational therapy, and physiotherapy. We defined art therapy as music, dance, and/or art (e.g., painting) therapy.

Readers interested in our analysis of the qualitative data may contact the first author.

## Results

Table 1 summarizes sociodemographic data of patients and staff.

**Table 1:** Sociodemographic data of patients and staff

Patients (*N* = 142)	Open setting (*n* = 74)	Locked setting (*n* = 68)	Differences
Gender (male/female)	32 female (43.2%)	30 female (44.1%)	χ² (1, *N* = 142) = 0.011, *p* = .916
Age (years)	*M* = 39 (*SD* = 13.8)	*M* = 38.8 (*SD* = 12.3)	*t*(140) = 0.081, *p* = .936
Primary education (years)	*M* = 11.3 (*SD* = 2.1)	*M* = 10.9 (*SD* = 2.4)	*t*(138) = 1.130, *p* = .260
Previous admissions to psychiatric hospital	*M* = 3 (*SD* = 1.1)	*M* = 3.1 (*SD* = 1.3)	*t*(138) = 0.554, *p* = .581
Primary diagnosis	-	-	-
Other Specified Mental Disorder Due to Another Medical Condition	1 (1.3%)	0	-
Substance-Induced Mental Disorders	0	3 (4.4%)	-
Schizophrenia Spectrum and Other Psychotic Disorders	65 (87.9%)	52 (76.5%)	-
Bipolar and Related Disorders	7 (9.5%)	11 (16.2%)	-
Depressive Disorders	1 (1.3%)	2 (2.9%)	-
Staff (*N* = 29)	Open setting (*n* = 13)	Locked setting (*n* = 16)	Differences
Profession	-	-	-
Medical doctor	1 (7.7%)	5 (31.3%)	-
Nurse	9 (69.2%)	9 (56.3%)	-
Psychologist	2 (15.4%)	1 (6.3%)	-
Social worker	1 (7.7%)	0	-
Medical assistant	0	1 (6.3%)	-
Work experience overall (years)	*M* = 15.5 (*SD* = 12.8)	*M* = 9.3 (*SD* = 6.1)	*t*(16.34) = 1.619, *p* = .125
Work experience on current ward (years)	*M* = 6.1 (*SD* = 6.6)	*M* = 4.3 (*SD* = 4.1)	*t*(27) = 0.874, *p* = .390

### Symptoms

#### Patients

Of the 142 patients who consented to participate, 138 completed the questionnaire on subjective symptom presence and relevance. The four patients who did not answer the questionnaire were being treated on locked wards. While 10 patients did not provide an answer for every symptom on the questionnaire, all but four patients (three on an open, one on a locked ward) rated at least one symptom as present. Five patients rated all 16 symptoms as present (see Table 2). Comparing open vs. locked ward settings, patients on the locked wards endorsed experiencing grandiosity (*M* = 0.54, *SD* = 0.50; *t*(126.305) = 3.105, *p* = .002, *d* = 0.53) and self-harming behavior (*M* = 0.38, *SD* = 0.49; *t*(120.963) = 2.435, *p* = .016, *d* = 0.42) significantly more often than patients on the open wards (*M*_*grandiosity*_ = 0.28, *SD* = 0.45; *M*_*self−harming behavior*_ = 0.19, *SD* = 0.39).

**Table 2:** Symptoms reported to be present by patients (*N* = 138) in descending order of frequency

Symptom	*n* (%)
Memory/attention problems	90 (63.4%)
Loneliness	82 (57.7%)
Lack of drive	81 (57%)
Depression	78 (54.9%)
Inability to think clearly	77 (54.2%)
Social anxiety	70 (49.3%)
Persecution	68 (47.9%)
Delusions	65 (45.8%)
Self-esteem	63 (44.4%)
Obsessions	61 (43%)
Aggression/anger	58 (40.8%)
Grandiosity	55 (38.7%)
Hearing voices	51 (35.9%)
Compulsions	41 (28.9%)
Self-harm	38 (26.8%)
Suicidal ideation	36 (25.4%)

**Fig. 1:**
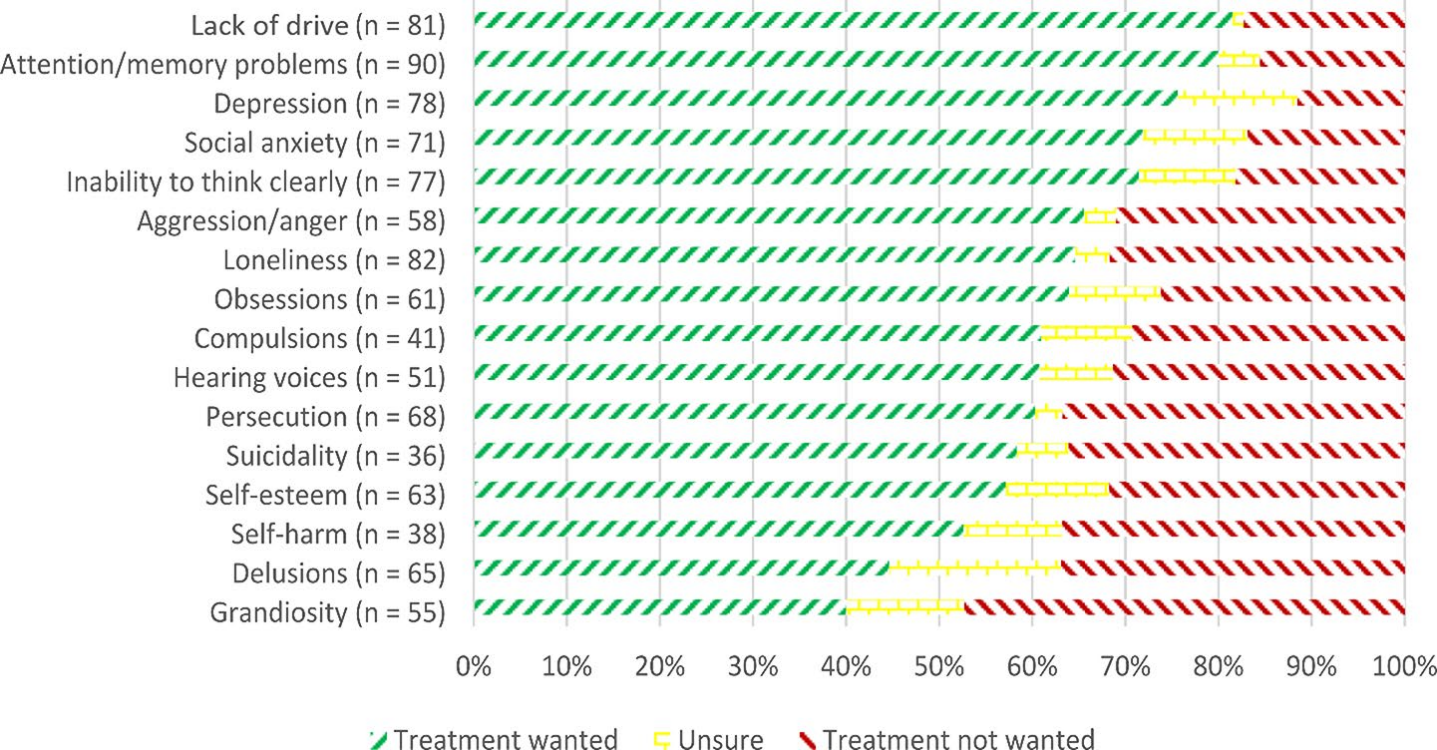
Subjective need for treatment of present symptoms as rated by patients

Figure 1 shows how many patients currently experiencing a given symptom wanted, did not want, or were unsure whether they wanted that symptom to be treated. There was no significant association of age or number of previous hospital admissions with the subjective need for treatment of any symptom.

To analyze the impact of symptom domains, setting and gender on the subjective need for treatment, we grouped the assessed symptoms into subscales based on theoretical assumptions (see Table S1 in Online Resource 1) and performed an ANOVA. The presence of symptoms varied considerably across patients, so we performed an ANOVA using the positive, affective, and neurocognitive symptom subscales as those were the three most frequently present subscales. Thus, 77 patients who had endorsed at least one symptom per subscale (positive, affective, and neurocognitive) as present were analyzed. A three-way ANOVA was performed to analyze the effect of symptom domains, treatment setting, and gender on whether the symptom domains should be treated. Due to the data violating assumptions of sphericity, ANOVA test statistics were estimated using the Huynh-Feldt method. There was a significant main effect of symptom domains on reported importance of treatment (*F*(1.844, 134.629) = 9.719, *p* < .001, η_p_² = 0.117), with neurocognitive symptoms being the most important for patients, followed by affective and positive symptoms. Bonferroni-corrected post-hoc tests showed that patients rated neurocognitive symptoms as significantly more important to treat (“should be treated”) than positive symptoms (*p* < .001) and affective symptoms (*p* = .047). However, patients did not rate affective symptoms as significantly more important to treat than positive symptoms (*p* = .157).

There was also a significant effect of setting on subjective need for treatment (*F*(1, 73) = 13.547, *p* < .001, η_p_² = 0.157), with patients on the open wards expressing a higher level of subjective need for treatment than patients on the locked wards. There was no significant main effect of gender (*F*(1, 73) = 0.026, *p* = .873, η_p_² < 0.001). There were no significant interactions between symptom domains by setting (*F*(1.844, 134.629) = 1.104, *p* = .331, η_p_² = 0.015), symptom domains by gender (*F*(1.844, 134.629) = 0.573, *p* = .552, η_p_² = 0.008), symptom domains by setting by gender (*F*(1.844, 134.629) = 1.572, *p* = .213, η_p_² = 0.021), or setting by gender (*F*(1, 73) = 0.228, *p* = .634, η_p_² = 0.003).

#### Staff

**Fig. 2:**
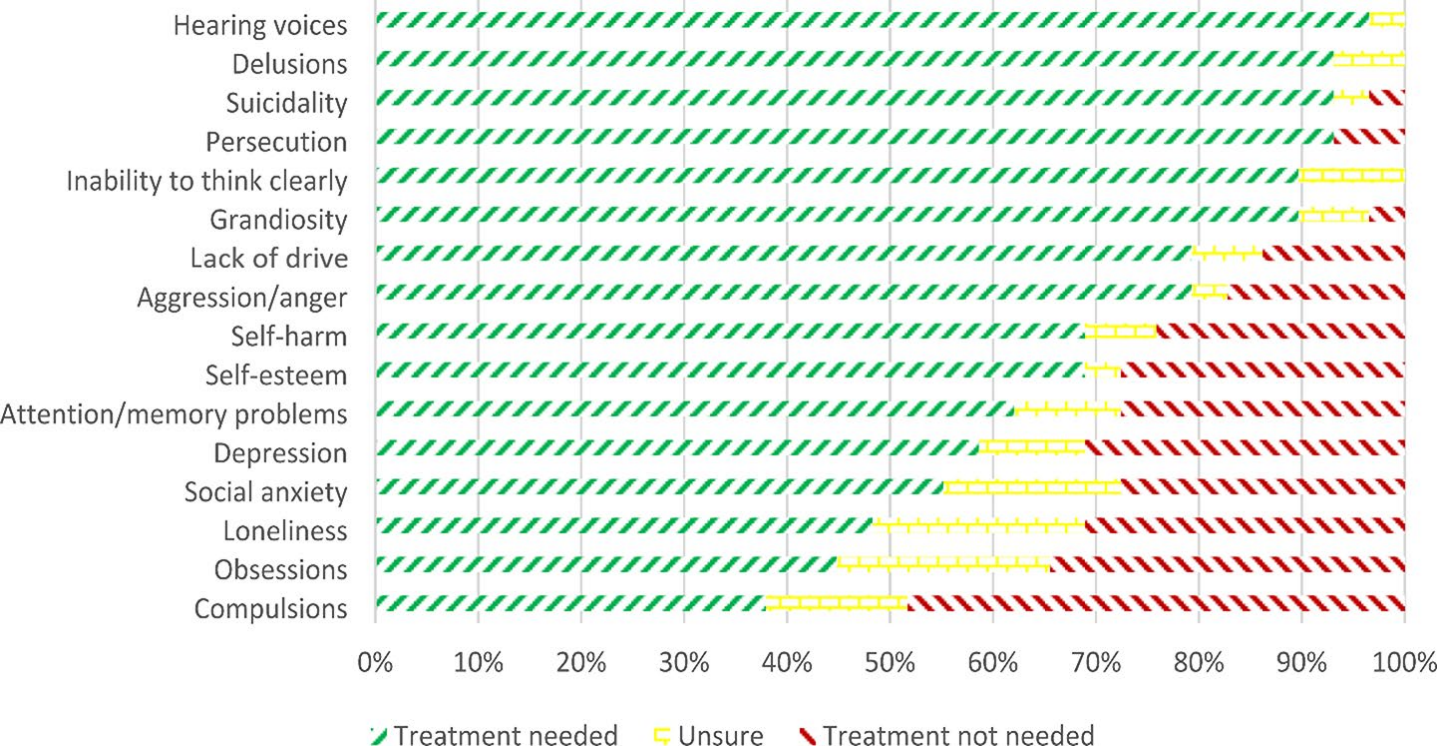
Need for treatment of present symptoms as rated by staff

Figure 2 shows how many staff members agreed with, were unsure about, or disagreed with the need to treat a given symptom on their ward. In parallel to the patient data analysis, positive, affective, and neurocognitive symptoms were compared in a two-way ANOVA analyzing the effect of symptom domains and treatment setting on staff’s view of treatment need. There was a significant main effect of symptom domains on treatment importance (*F*(2,54) = 30.059, *p* < .001, η_p_² = 0.527). Bonferroni-corrected post-hoc tests showed that staff viewed positive symptoms as significantly more important to treat than neurocognitive symptoms (*p* < .001) and than affective symptoms (*p* < .001). Staff also rated neurocognitive symptoms as significantly more treatment worthy than affective symptoms (*p* = .009). There was a significant effect of treatment setting on treatment importance, *F*(1,27) = 10.902, *p* = .003, η_p_² = 0.288, in that staff on open wards endorsed a higher need for treatment of symptoms than staff on locked wards. However, there was no significant type of symptom by setting interaction, *F*(2,54) = 0.841, *p* = .437, η_p_² = 0.030.

### Treatment Preferences

#### Patients

The numbers of patients familiar with and desiring various treatments are shown in Table 3. The largest percentage of patients endorsed wanting individual psychotherapy (*n* = 107; 82.9%), while the smallest percentage endorsed group psychotherapy (*n* = 72; 54.5%). Thus, all treatments were desired by more than 50% of patients.

**Table 3:** Patients’ familiarity with various treatments and patients’ and staffs’ endorsement of these treatments

Treatment	Known by patients		Desired by patients				Endorsed by staff (*n* = 28)		
	*n*	yes (%)	*n*	yes (%)	no (%)	unsure (%)	yes (%)	no (%)	unsure (%)
Individual psychotherapy	141	130 (91.5%)	129	107 (82.9%)	16 (12.4%)	6 (4.7%)	26 (92.9%)	0	2 (7.1%)
Physiotherapy	139	134 (94.4%)	134	107 (79.9%)	23 (17.2%)	4 (3.0%)	28 (100%)	0	0
Art therapy	140	122 (85.9%)	122	92 (75.4%)	27 (22.1%)	3 (2.5%)	23 (82.1%)	0	5 (17.9%)
Occupational therapy	141	134 (94.4%)	134	100 (74.6%)	30 (22.4%)	4 (3.0%)	28 (100%)	0	0
Mindfulness	141	88 (62.0%)	87	58 (66.7%)	23 (26.4%)	6 (6.9%)	17 (60.7%)	3 (10.7%)	8 (28.6%)
Medication	139	131 (92.3%)	131	84 (64.1%)	41 (31.3%)	6 (4.6%)	28 (100%)	0	0
Psychoeducation	140	81 (57.0%)	81	47 (58.0%)	28 (34.6%)	6 (7.4%)	21 (75%)	2 (7.1%)	5 (17.9%)
Group psychotherapy	140	132 (93.0%)	132	72 (54.5%)	51 (38.6%)	9 (6.8%)	22 (78.6%)	1 (3.6%)	5 (17.9%)

To further compare patients’ appraisal of different treatments, we performed a three-way ANOVA analyzing the effect of treatment type, setting, and gender on appraisal of treatment. As all treatments except for mindfulness and psychoeducation were known by more than 85% of patients, we excluded mindfulness and psychoeducation. Altogether, 82 patients knew of all other six types of treatments and were included in the analysis. Due to the data violating assumptions of sphericity, ANOVA test statistics were estimated using the Huynh-Feldt method. There was a significant main effect of treatment type on appraisal of treatment (*F*(4.725, 368.535) = 6.340, *p* < .001, η_p_² = 0.075). Bonferroni-corrected post-hoc tests showed that patients preferred individual psychotherapy over medication (*p* = .005) and over group psychotherapy (*p* < .001). They also preferred physiotherapy significantly over group psychotherapy (*p* < .001). There were also statistically significant interaction effects of treatment type by setting (*F*(4.725, 368.535) = 2.814, *p* = .019, η_p_² = 0.035) and of treatment type by gender (*F*(4.725, 368.535) = 5.841, *p* < .001, η_p_² = 0.070). Patients on the open wards endorsed medication more than patients on the locked wards. Women endorsed occupational therapy, art therapy, and physiotherapy more than men, while men endorsed medication more than women. Neither the main effect of setting (*F*(1, 78) = 3.383, *p* = .070, η_p_² = 0.042), nor the main effect of gender (*F*(1, 78) = 1.412, *p* = .238, η_p_² = 0.018), the interaction effect of setting by gender (*F*(1, 78) = 2.632, *p* = .109, η_p_² = 0.033), nor the three-way interaction of treatment type by setting by gender (*F*(4.725, 368.535) = 0.636, *p* = .663, η_p_² = 0.008) on treatment preference were statistically significant.

#### Staff

Staff endorsements of which treatments patients should receive can be found in Table 3. We performed a two-way repeated measures ANOVA analyzing the effect of treatment type and treatment setting on staff’s perceived indication for treatment. In parallel to the patient data analysis, we left mindfulness and psychoeducation out of the ANOVA. There was a significant main effect of treatment type on indication for treatment (*F*(5, 130) = 3.902, *p* = .003, η_p_² = 0.130). Bonferroni-corrected post-hoc tests showed that staff endorsed physiotherapy, occupational therapy, and medication significantly more than group psychotherapy (each *p* = .022). There was no significant main effect of setting (*F*(1, 26) = 0.572, *p* = .456, η_p_² = 0.022) nor significant treatment type by setting interaction (*F*(5, 130) = 0.230, *p* = .949, η_p_² = 0.009).

## Discussion

In the present study, we examined which symptoms patients with acute psychosis reported experiencing while being treated on locked and on open psychiatric wards, which of their symptoms they believed should be treated, and which treatment options patients would like to receive. In addition, we reported which symptoms staff on the same wards believed should be treated in patients with psychosis in their setting and which treatments staff believed patients should receive.

Patients regarded the need for treatment of neurocognitive symptoms as significantly greater than the need for treatment of affective or positive symptoms. Staff considered the need for treatment of positive symptoms as significantly greater than for the other symptom domains, followed by neurocognitive symptoms, to which they assigned a significantly higher need for treatment than to affective symptoms. Across symptoms, both patients and staff on open wards saw greater treatment need than patients and staff on locked wards.

All treatment types were desired by more than half of all patients. Endorsement of psychopharmacological treatment differed between settings, with patients on open wards showing higher endorsement than patients on locked wards. While all staff endorsed that their patients should receive occupational therapy, medication, and physiotherapy, endorsement of the remaining therapies ranged from 93% for individual psychotherapy to 61% for mindfulness. There were no differences in staff’s endorsements of treatments between the open and locked settings. The largest difference in agreement between patients and staff regarding a specific treatment was found for medication, which was endorsed by all staff (100%) but only by approximately two thirds (64.1%) of patients. Patients also endorsed occupational therapy (74.6%) and group psychotherapy (54.5%) less than staff did (100% endorsement for occupational therapy and 78.6% endorsement for group psychotherapy). All other therapies except mindfulness-based interventions were endorsed more by staff than by patients, but by a lesser extent. Overall, physiotherapy and individual psychotherapy were endorsed most frequently by patients and also received some of the highest rankings from staff, while psychoeducation and group psychotherapy were among the three lowest ranked therapy options for both patients and staff.

Our results add to a growing body of literature that shows the importance of affective and neurocognitive symptoms to patients [20, 21, 26]. When directly compared, patients’ subjective need for treatment for these symptoms was higher than for positive symptoms. While it has long been argued that positive symptoms must inherently be the primary target of therapy, patients have previously reported that they do not necessarily view all aspects of their positive symptoms as negative [19]. Goal formulations for cognitive behavioral therapy for psychosis also show that patients engaging in therapy often have other targets in addition to symptom reduction [27]. One reason why staff may focus on reducing positive symptoms as a treatment target is that antipsychotic medication is the most effective intervention at reducing these symptoms but is much less effective at improving negative or neurocognitive symptoms [28]. Another important argument for prioritizing the treatment of positive symptoms is that psychotic symptoms, particularly positive symptoms, constitute a risk marker for suicidal thoughts and behaviors. Positive symptoms such as persecutory ideation and auditory hallucinations have also been associated in one study with suicidal thoughts and attempts in community samples [29], although the authors also found that affective symptoms such as depression mediated the associations between positive symptoms and suicidality. In addition, studies on various populations show that both positive symptoms and affective symptoms such as depression are associated with general distress in patients with psychosis [30, 31]. Thus, targeting affective symptoms such as depression during the acute phase of illness may be worthwhile, and clinicians should first assess the harmfulness of a given symptom before determining its treatment priority [13] and should take patients’ subjective needs into consideration. Some symptoms characterized as affective, such as lack of drive, may also be induced by antipsychotic medication [32]. Lack of drive, if present, was the symptom patients most often wanted treatment for. This may also relate to many patients’ not wishing to receive medication as treatment because it might either aggravate an existing lack of drive or induce it when it had not been present previously. As neurocognitive impairments are associated with a range of adverse outcomes such as poorer functional outcome and more negative and disorganized symptoms [33], and both patients and staff rate them as important treatment targets, interventions addressing these difficulties directly should also be incorporated into the acute care setting.

Our findings demonstrate that while both patients and staff deem certain symptoms important to treat and the two groups show strong agreement on some interventions, there are also differences in preferences between these two groups. This may be due to differing models and conceptualizations of mental health and illness, such as more biologically oriented views that may be more common among medical staff and views related more to a personal recovery orientation that may be more common among patients [15, 16]. People with psychosis who feel supported in their recovery process and who report higher shared decision making show higher treatment satisfaction [34, 35], which is a commonly used quality indicator of mental health care [36]. In a long-term involuntary treatment setting, the consideration of patients’ opinions was also a strong predictor of treatment satisfaction and of subjective quality of life [37]. Thus, taking patients’ treatment preferences into account more may also contribute to improving acute involuntary inpatient care. The results of this study may help clinicians realize that patients may have treatment priorities different from clinicians’, compare individual patients’ priorities with the priorities of other patients with the same disorder, and normalize seemingly unconventional priorities such as art therapy among patients and staff.

### Limitations

The present study is subject to several limitations. Not all patients were experiencing all symptoms nor were familiar with all assessed treatments, resulting in different sample sizes across analyses. The low number of staff members recruited also limits the generalizability. Specifically, we could not examine potential differences between professions regarding their treatment preferences as almost two thirds of the staff sample were nurses and samples sizes for other professions were too small to allow meaningful analyses. Potential differences in staffs’ preferences may be caused by different illness models that members of various professions base their understanding of treatment on [15], but this topic could not be analyzed here. This should be considered in more depth in future research. In addition, we used a self-report measure to assess the presence of symptoms. For the assessment of delusion-related symptoms (e.g., delusions of persecution or grandiosity), the symptoms were not named directly but were instead described indirectly (e.g., “Is there anything special about you? Do you have any special abilities or powers?” for grandiosity). This was done deliberately to assess which symptoms patients themselves viewed as present. Because positive symptoms, in particular, are often not recognized by patients as part of their illness, we were aware that even if a patient agreed that they felt they were being prosecuted or that they had extraordinary powers and a unique calling to fulfill, this did not necessarily mean that the patient viewed their experience as a symptom. It is also possible that patients deliberately denied experiencing certain symptoms, particularly delusion-related symptoms, as these are highly stigmatized and patients may be suspicious of the interviewer or afraid to disclose such symptoms for fear of consequences (e.g., augmentation of antipsychotic medication). Therefore, the number of patients experiencing positive symptoms may be underestimated due to the self-report format. We suggest that future studies include both self-reported and clinician-rated symptoms to compare self- and other ratings, particularly in the locked ward setting. This would also enable researchers to distinguish in more detail whether patients are subjectively suffering from certain positive symptoms and thus may be open to treatment for them even if they themselves do not identify these experiences as symptoms of an illness. We also deliberately used a simplified questionnaire to assess patients’ treatment knowledge and preferences to accommodate patients’ limited capacity to concentrate on the assessment while experiencing acute symptoms. Thus, nuances between different art therapies (e.g., music vs. dance) and between different types of individual or group psychotherapies cannot be discerned from this research design and should be investigated in more detail in the future. In addition, future research should specifically recruit patients with psychosis who are experiencing certain symptoms such as self-harming behavior to assess these symptoms’ relevance in comparison to positive, neurocognitive, and affective symptoms. Furthermore, patients’ medication at the time of the assessment should be recorded systematically and assessed for its potential influence on patients’ treatment priorities for specific symptoms since medication itself can cause and/or exacerbate symptoms, such as lack of drive, that patients want to alleviate.

### Clinical Implications

While it is often assumed that patients who experience acute psychosis have no insight into their symptoms and therefore no motivation for treatment, we found that this is not generally true. Clinicians who work in locked psychiatric care settings should focus on addressing neurocognitive and affective difficulties as well as positive symptoms with their patients to engage them in the therapeutic process. Neurocognitive symptoms, in particular, were ranked as high treatment priorities by both patients and staff, yet treatment options for these symptoms, particularly in the acute setting, are thus far mostly limited to medication. More psychosocial interventions targeting neurocognitive symptoms should be developed and assessed for their acceptability and effectiveness with people experiencing acute psychosis. Staff should also consider that patients want a variety of treatment options and offer more interventions to motivate patients to actively participate in an overarching biopsychosocial treatment plan. This would contribute to meeting patients’ increasingly voiced desire for more psychosocial treatment options [38] and might address the need that both patients and staff in inpatient settings report for the treatment of psychosis beyond symptom reduction [13]. Our results can inform shared decision making and goal formulation processes, which are essential to forming a constructive working alliance and fostering motivation for change in patients.

### Conclusion

Both patients and staff on acute wards see much room for improvement of the therapeutic framework employed on such wards. Our study adds to previous findings that even patients with severe acute psychotic symptoms recognize the need to work on certain symptoms and are open to receiving a range of treatments for them. Thus, the next challenge will be to develop and evaluate more psychosocial treatment options specifically designed for the acute setting to improve patients’ experience and to contribute to stabilization during their stay on the ward. Greater consideration of patients’ preferences for their own treatment may help with goal formulation, establishing a therapeutic relationship, fostering motivation for change, and thus long-term stabilization.

## Electronic Supplementary Material

Below is the link to the electronic supplementary material.

Supplementary Material 1

## Data Availability

The data that support the findings of this study are available from the corresponding author upon reasonable request.
