# Correction: Treatment Preferences in Acute Psychosis: A Comparison of Patient and Staff Perspectives on Symptom Prioritization and Biopsychosocial Interventions

**DOI:** 10.1007/s11126-024-10107-5

**Published:** 2024-11-29

**Authors:** Rabea Fischer, Steffen Moritz, Jakob Scheunemann, Matthias Nagel, Charlotte Osthues, Daniel Schöttle, Daniel Luedecke

**Affiliations:** 1 Department of Psychiatry and Psychotherapy, University Medical Center Hamburg-Eppendorf, Hamburg, Germany; 2 Department of Psychiatry and Psychotherapy, Asklepios Clinic North-Wandsbek, Hamburg, Germany; 3 Clinic for Psychiatry and Psychotherapy, University Clinic Schleswig-Holstein, Campus Luebeck, Luebeck, Germany; 4 Department of Psychiatry and Psychotherapy, Asklepios Clinic Harburg, Hamburg, Germany

**Psychiatric Quarterly**

10.1007/s11126-024-10099-2

The original version of this paper contains mistakes. First, there were words that needed to be updated to complete the sentence. These errors have been described below. Second, the reference entries were presented incorrectly and should have been formatted according to Vancouver reference style.

Data Analysis: in line "... within-subject factor and ward setting as between-subject..." the word "ward" has been added before setting.

Discussion: in line "... paper may help clinicians realize that patients may have..." should have been "... study may help clinicians realize that patients may have..."

The original article has been corrected.

