# Supplementary material for: Treatment Preferences in Acute Psychosis: A Comparison of Patient and Staff Perspectives on Symptom Prioritization and Biopsychosocial Interventions
Source: Psychiatr Q. 2024 Oct 29;96(1):1–16. doi: 10.1007/s11126-024-10099-2 (PMC11929731; doi:10.1007/s11126-024-10099-2)
Supplement: Supplementary file 1 — Supplementary Material 1 [file 11126_2024_10099_MOESM1_ESM.docx]

**Supplementary Material**

**Table S1. Subscales of symptoms and presence in patients**

| Subscale | **Items** | Number (%) of patients who endorsed at least one item per scale as present |
| --- | --- | --- |
| Positive | grandiosity, hearing voices, persecution, delusions | 105 (76.1%) |
| Affective | listlessness, depression, loneliness, self-esteem, social anxiety | 111 (80.4%) |
| Cognitive | memory and attention, ability to think clearly | 100 (72.5%) |
| Obsessive-compulsive | obsessions, compulsions | 69 (50%) |
| Self-harm | self-harming, suicidal thoughts | 51 (37%) |
| Aggression/anger | Aggression/anger | 58 (40.8%) |

**Figures**

**Figure S1.** *Flow chart of participant inclusion.*

|  | ***N* = 1985 screened** |  |
| --- | --- | --- |
|  |  |  |
| **Open ward setting, *N* = 492** |  | **Locked ward setting, *N* = 1493** |
|  |  |  |
| Met exclusion criteria (*n* = 45) |  | Met exclusion criteria (*n* = 237) |
|  |  |  |
|  |  | Symptom load too high to take part in the survey (*n* = 68) |
|  |  |  |
| Lack of German (*n* = 25) |  | Lack of German (*n* = 46) |
|  |  |  |
| Ward staff advised against inclusion (*n* = 14) |  | Ward staff advised against inclusion (*n* = 34) |
|  |  |  |
| Intellectual disability (*n* = 6) |  | Intellectual disability (*n* = 10) |
|  |  |  |
|  |  | Dementia (*n* = 79) |
|  |  |  |
| Refused participation (*n* = 61) |  | Refused participation (*n* = 102) |
|  |  |  |
| Released from ward before they could be approached (*n* = 118) |  | Released from ward before they could be approached (*n* = 453) |
|  |  |  |
| Transferred to another ward before they could be approached (*n* = 35) |  | Transferred to another ward before they could be approached (*n* = 392) |
|  |  |  |
| Recruitment break due to Covid-19 (*n* = 109) |  | Recruitment break due to Covid-19 (*n* = 178) |
|  |  |  |
| Other reasons (*n* = 50) |  | Other reasons (*n* = 63) |
|  |  |  |
| **Included in final sample (*n* = 74)** |  | **Included in final sample (*n* = 68)** |
|  |  |  |
|  | ***N* = 142 Final sample** |  |
